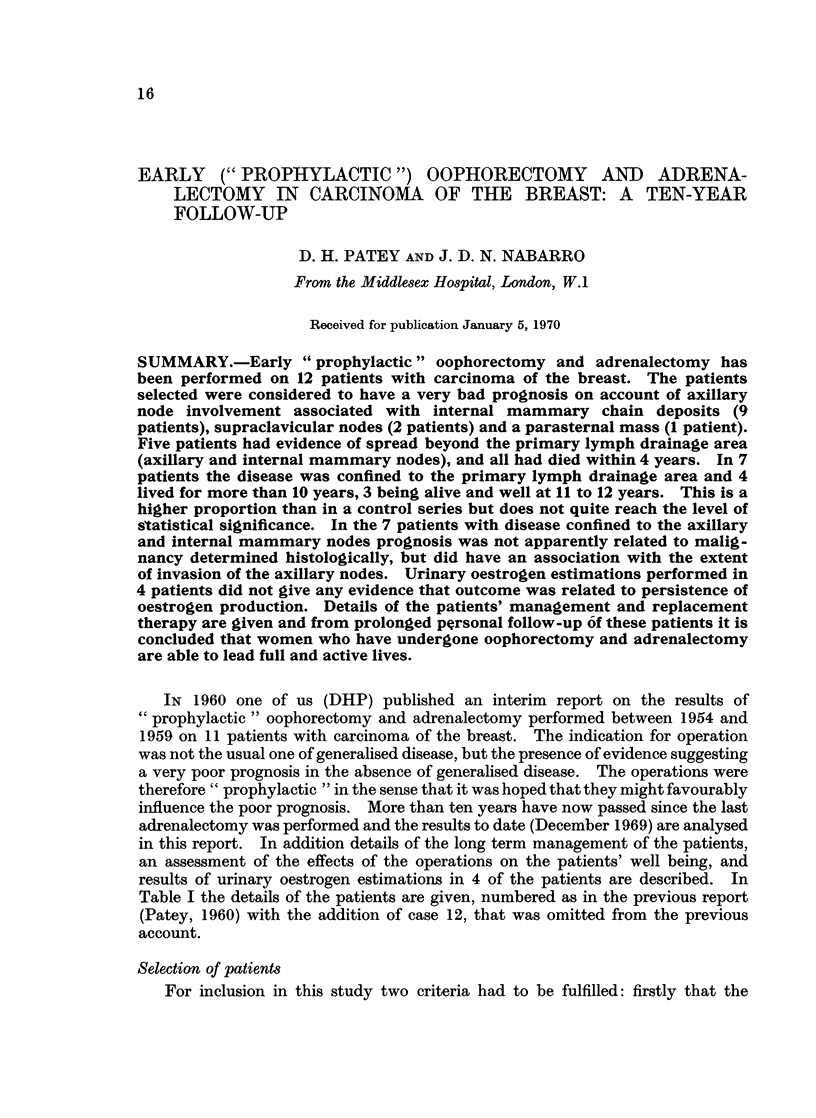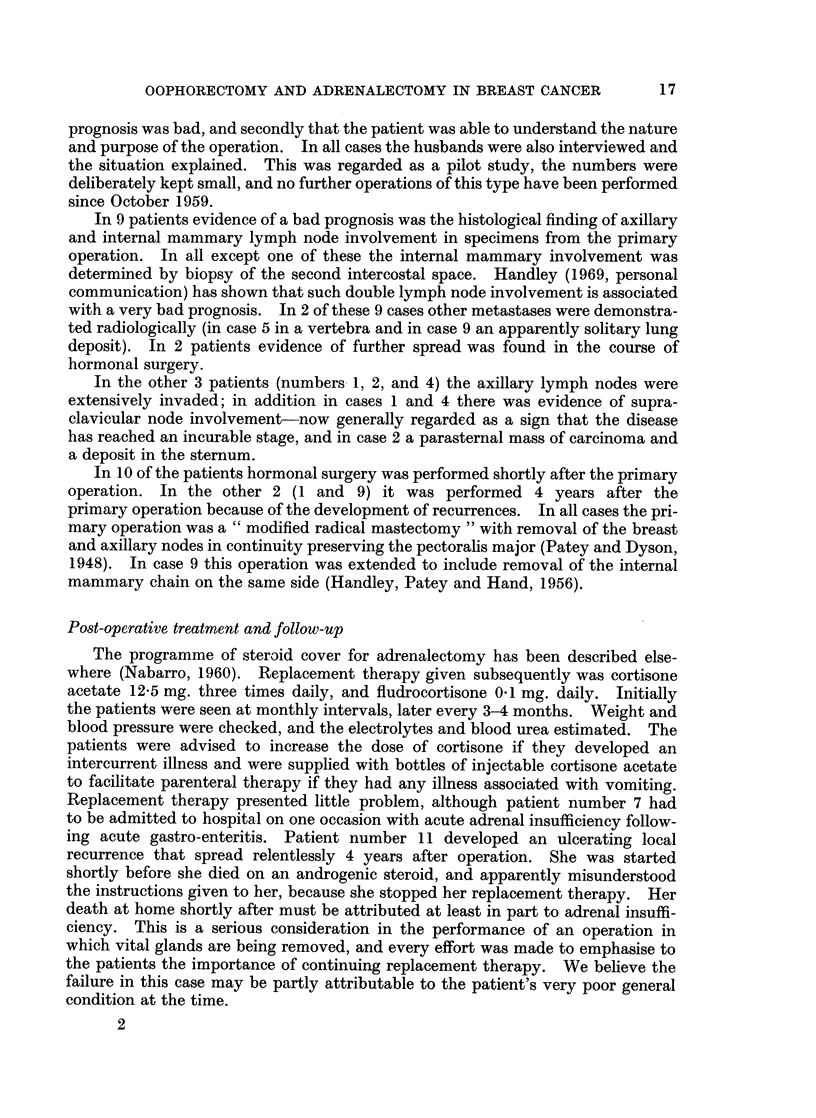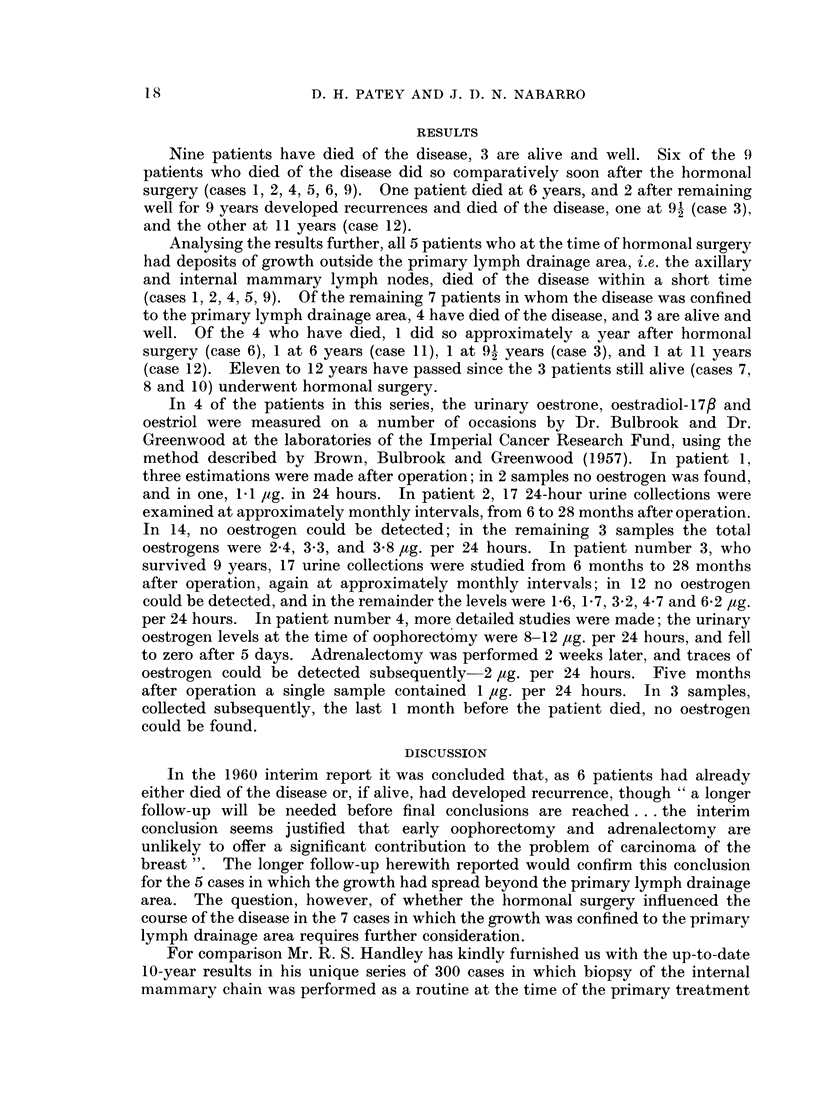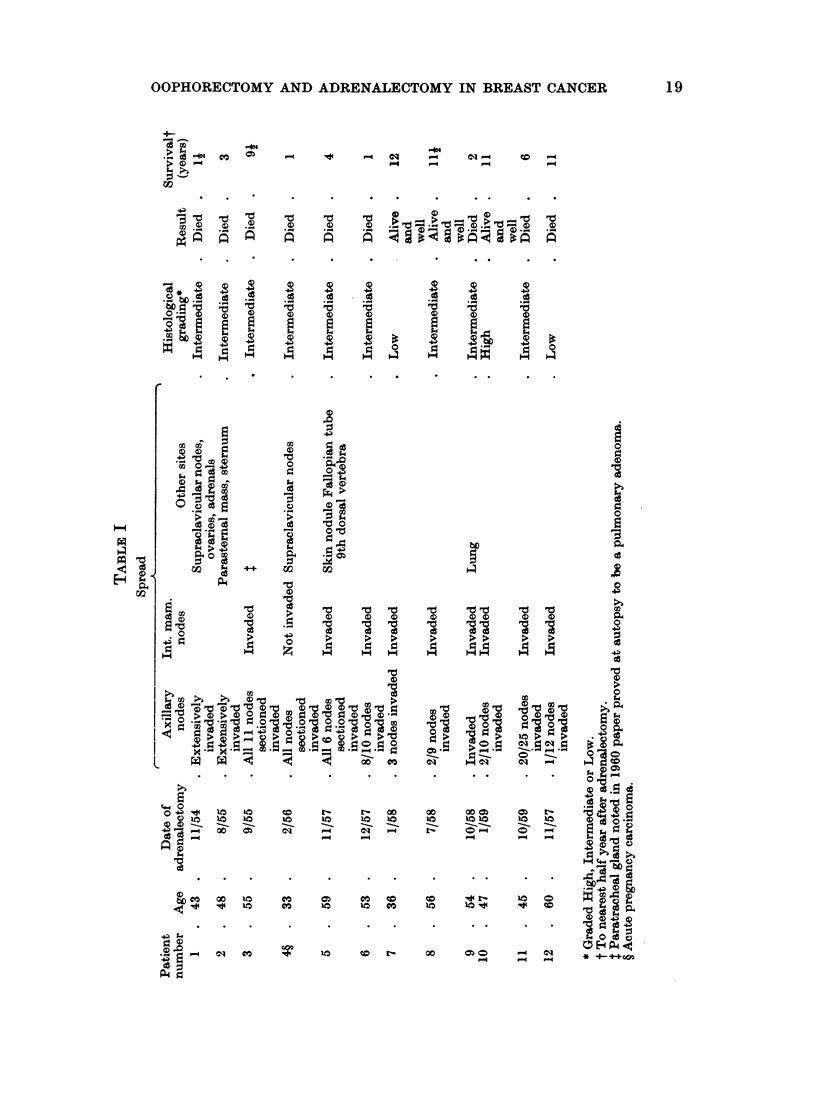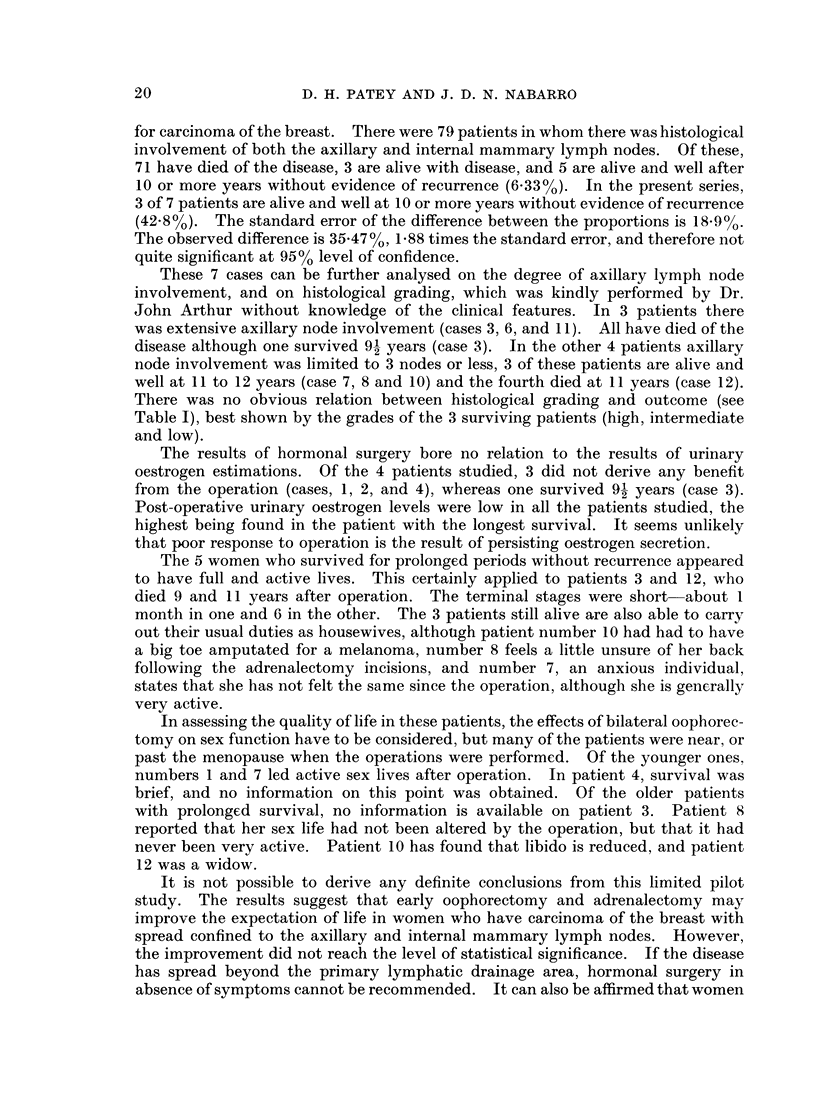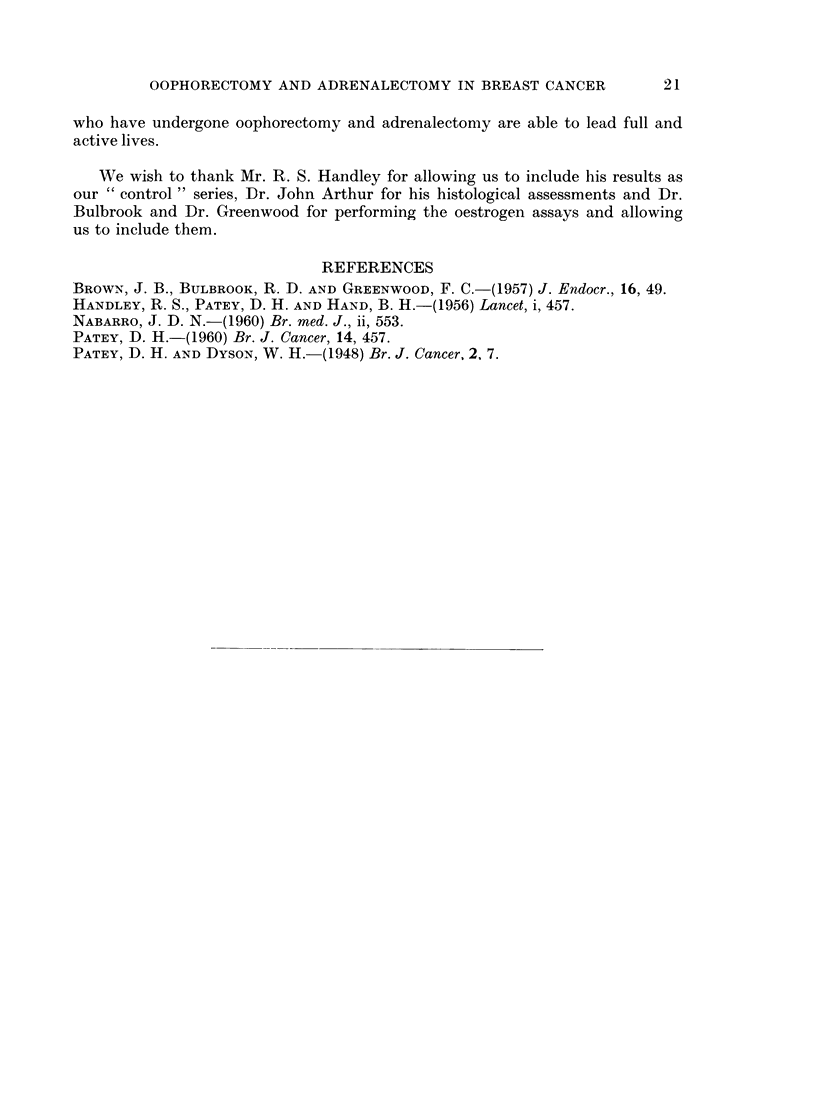# Early (“Prophylactic”) Oophorectomy and Adrenalectomy in Carcinoma of the Breast: A Ten-Year Follow-Up

**DOI:** 10.1038/bjc.1970.3

**Published:** 1970-03

**Authors:** D. H. Patey, J. D. N. Nabarro

## Abstract

Early “prophylactic” oophorectomy and adrenalectomy has been performed on 12 patients with carcinoma of the breast. The patients selected were considered to have a very bad prognosis on account of axillary node involvement associated with internal mammary chain deposits (9 patients), supraclavicular nodes (2 patients) and a parasternal mass (1 patient). Five patients had evidence of spread beyond the primary lymph drainage area (axillary and internal mammary nodes), and all had died within 4 years. In 7 patients the disease was confined to the primary lymph drainage area and 4 lived for more than 10 years, 3 being alive and well at 11 to 12 years. This is a higher proportion than in a control series but does not quite reach the level of statistical significance. In the 7 patients with disease confined to the axillary and internal mammary nodes prognosis was not apparently related to malignancy determined histologically, but did have an association with the extent of invasion of the axillary nodes. Urinary oestrogen estimations performed in 4 patients did not give any evidence that outcome was related to persistence of oestrogen production. Details of the patients' management and replacement therapy are given and from prolonged personal follow-up of these patients it is concluded that women who have undergone oophorectomy and adrenalectomy are able to lead full and active lives.


### EARLY ("PROPHYLACTIC") OOPHORECTOMY AND ADRENALECTOMY IN CARCINOMA OF THE BREAST: A TEN-YEAR FOLLOW-UP

D. H. PATEY AND J. D. N. NABARRO

*From the Middlesex Hospital, London, W.1*

Received for publication January 5, 1970

**SUMMARY.—Early "prophylactic" oophorectomy and adrenalectomy has been performed on 12 patients with carcinoma of the breast. The patients selected were considered to have a very bad prognosis on account of axillary node involvement associated with internal mammary chain deposits (9 patients), supraclavicular nodes (2 patients) and a parasternal mass (1 patient). Five patients had evidence of spread beyond the primary lymph drainage area (axillary and internal mammary nodes), and all had died within 4 years. In 7 patients the disease was confined to the primary lymph drainage area and 4 lived for more than 10 years, 3 being alive and well at 11 to 12 years. This is a higher proportion than in a control series but does not quite reach the level of statistical significance. In the 7 patients with disease confined to the axillary and internal mammary nodes prognosis was not apparently related to malignancy determined histologically, but did have an association with the extent of invasion of the axillary nodes. Urinary oestrogen estimations performed in 4 patients did not give any evidence that outcome was related to persistence of oestrogen production. Details of the patients' management and replacement therapy are given and from prolonged personal follow-up of these patients it is concluded that women who have undergone oophorectomy and adrenalectomy are able to lead full and active lives.**

In 1960 one of us (DHP) published an interim report on the results of "prophylactic" oophorectomy and adrenalectomy performed between 1954 and 1959 on 11 patients with carcinoma of the breast. The indication for operation was not the usual one of generalised disease, but the presence of evidence suggesting a very poor prognosis in the absence of generalised disease. The operations were therefore "prophylactic" in the sense that it was hoped that they might favourably influence the poor prognosis. More than ten years have now passed since the last adrenalectomy was performed and the results to date (December 1969) are analysed in this report. In addition details of the long term management of the patients, an assessment of the effects of the operations on the patients' well being, and results of urinary oestrogen estimations in 4 of the patients are described. In Table I the details of the patients are given, numbered as in the previous report (Patey, 1960) with the addition of case 12, that was omitted from the previous account.

*Selection of patients*

For inclusion in this study two criteria had to be fulfilled: firstly that the prognosis was bad, and secondly that the patient was able to understand the nature and purpose of the operation. In all cases the husbands were also interviewed and the situation explained. This was regarded as a pilot study, the numbers were deliberately kept small, and no further operations of this type have been performed since October 1959.

In 9 patients evidence of a bad prognosis was the histological finding of axillary and internal mammary lymph node involvement in specimens from the primary operation. In all except one of these the internal mammary involvement was determined by biopsy of the second intercostal space. Handley (1969, personal communication) has shown that such double lymph node involvement is associated with a very bad prognosis. In 2 of these 9 cases other metastases were demonstrated radiologically (in case 5 in a vertebra and in case 9 an apparently solitary lung deposit). In 2 patients evidence of further spread was found in the course of hormonal surgery.

In the other 3 patients (numbers 1, 2, and 4) the axillary lymph nodes were extensively invaded; in addition in cases 1 and 4 there was evidence of supraclavicular node involvement—now generally regarded as a sign that the disease has reached an incurable stage, and in case 2 a parasternal mass of carcinoma and a deposit in the sternum.

In 10 of the patients hormonal surgery was performed shortly after the primary operation. In the other 2 (1 and 9) it was performed 4 years after the primary operation because of the development of recurrences. In all cases the primary operation was a " modified radical mastectomy " with removal of the breast and axillary nodes in continuity preserving the pectoralis major (Patey and Dyson, 1948). In case 9 this operation was extended to include removal of the internal mammary chain on the same side (Handley, Patey and Hand, 1956).

*Post-operative treatment and follow-up*

The programme of steroid cover for adrenalectomy has been described elsewhere (Nabarro, 1960). Replacement therapy given subsequently was cortisone acetate 12·5 mg. three times daily, and fludrocortisone 0·1 mg. daily. Initially the patients were seen at monthly intervals, later every 3–4 months. Weight and blood pressure were checked, and the electrolytes and blood urea estimated. The patients were advised to increase the dose of cortisone if they developed an intercurrent illness and were supplied with bottles of injectable cortisone acetate to facilitate parenteral therapy if they had any illness associated with vomiting. Replacement therapy presented little problem, although patient number 7 had to be admitted to hospital on one occasion with acute adrenal insufficiency following acute gastro-enteritis. Patient number 11 developed an ulcerating local recurrence that spread relentlessly 4 years after operation. She was started shortly before she died on an androgenic steroid, and apparently misunderstood the instructions given to her, because she stopped her replacement therapy. Her death at home shortly after must be attributed at least in part to adrenal insufficiency. This is a serious consideration in the performance of an operation in which vital glands are being removed, and every effort was made to emphasise to the patients the importance of continuing replacement therapy. We believe the failure in this case may be partly attributable to the patient's very poor general condition at the time.

### RESULTS

Nine patients have died of the disease, 3 are alive and well. Six of the 9 patients who died of the disease did so comparatively soon after the hormonal surgery (cases 1, 2, 4, 5, 6, 9). One patient died at 6 years, and 2 after remaining well for 9 years developed recurrences and died of the disease, one at $9\frac{1}{2}$ (case 3), and the other at 11 years (case 12).

Analysing the results further, all 5 patients who at the time of hormonal surgery had deposits of growth outside the primary lymph drainage area, *i.e.* the axillary and internal mammary lymph nodes, died of the disease within a short time (cases 1, 2, 4, 5, 9). Of the remaining 7 patients in whom the disease was confined to the primary lymph drainage area, 4 have died of the disease, and 3 are alive and well. Of the 4 who have died, 1 did so approximately a year after hormonal surgery (case 6), 1 at 6 years (case 11), 1 at $9\frac{1}{2}$ years (case 3), and 1 at 11 years (case 12). Eleven to 12 years have passed since the 3 patients still alive (cases 7, 8 and 10) underwent hormonal surgery.

In 4 of the patients in this series, the urinary oestrone, oestradiol-17$\beta$ and oestriol were measured on a number of occasions by Dr. Bulbrook and Dr. Greenwood at the laboratories of the Imperial Cancer Research Fund, using the method described by Brown, Bulbrook and Greenwood (1957). In patient 1, three estimations were made after operation; in 2 samples no oestrogen was found, and in one, 1·1 $\mu$g. in 24 hours. In patient 2, 17 24-hour urine collections were examined at approximately monthly intervals, from 6 to 28 months after operation. In 14, no oestrogen could be detected; in the remaining 3 samples the total oestrogens were 2·4, 3·3, and 3·8 $\mu$g. per 24 hours. In patient number 3, who survived 9 years, 17 urine collections were studied from 6 months to 28 months after operation, again at approximately monthly intervals; in 12 no oestrogen could be detected, and in the remainder the levels were 1·6, 1·7, 3·2, 4·7 and 6·2 $\mu$g. per 24 hours. In patient number 4, more detailed studies were made; the urinary oestrogen levels at the time of oophorectomy were 8–12 $\mu$g. per 24 hours, and fell to zero after 5 days. Adrenalectomy was performed 2 weeks later, and traces of oestrogen could be detected subsequently—2 $\mu$g. per 24 hours. Five months after operation a single sample contained 1 $\mu$g. per 24 hours. In 3 samples, collected subsequently, the last 1 month before the patient died, no oestrogen could be found.

### DISCUSSION

In the 1960 interim report it was concluded that, as 6 patients had already either died of the disease or, if alive, had developed recurrence, though " a longer follow-up will be needed before final conclusions are reached . . . the interim conclusion seems justified that early oophorectomy and adrenalectomy are unlikely to offer a significant contribution to the problem of carcinoma of the breast ". The longer follow-up herewith reported would confirm this conclusion for the 5 cases in which the growth had spread beyond the primary lymph drainage area. The question, however, of whether the hormonal surgery influenced the course of the disease in the 7 cases in which the growth was confined to the primary lymph drainage area requires further consideration.

For comparison Mr. R. S. Handley has kindly furnished us with the up-to-date 10-year results in his unique series of 300 cases in which biopsy of the internal mammary chain was performed as a routine at the time of the primary treatment

TABLE I

| Patient number | Age | Date of adrenalectomy | Spread: Axillary nodes | Spread: Int. mam. nodes | Spread: Other sites | Histological grading* | Result | Survival† (years) |
|---|---|---|---|---|---|---|---|---|
| 1 | 43 | 11/54 | Extensively invaded | | Supraclavicular nodes, ovaries, adrenals | Intermediate | Died | 1½ |
| 2 | 48 | 8/55 | Extensively invaded | | Parasternal mass, sternum | Intermediate | Died | 3 |
| 3 | 55 | 9/55 | All 11 nodes sectioned invaded | Invaded | ‡ | Intermediate | Died | 9½ |
| 4§ | 33 | 2/56 | All nodes sectioned invaded | Not invaded | Supraclavicular nodes | Intermediate | Died | 1 |
| 5 | 59 | 11/57 | All 6 nodes sectioned invaded | Invaded | Skin nodule Fallopian tube 9th dorsal vertebra | Intermediate | Died | 4 |
| 6 | 53 | 12/57 | 8/10 nodes invaded | Invaded | | Intermediate | Died | 1 |
| 7 | 36 | 1/58 | 3 nodes invaded | Invaded | | Low | Alive and well | 12 |
| 8 | 56 | 7/58 | 2/9 nodes invaded | Invaded | | Intermediate | Alive and well | 11½ |
| 9 | 54 | 10/58 | Invaded | Invaded | Lung | Intermediate | Died | 2 |
| 10 | 47 | 1/59 | 2/10 nodes invaded | Invaded | | High | Alive and well | 11 |
| 11 | 45 | 10/59 | 20/25 nodes invaded | Invaded | | Intermediate | Died | 6 |
| 12 | 60 | 11/57 | 1/12 nodes invaded | Invaded | | Low | Died | 11 |

* Graded High, Intermediate or Low.

† To nearest half year after adrenalectomy.

‡ Paratracheal gland noted in 1960 paper proved at autopsy to be a pulmonary adenoma.

§ Acute pregnancy carcinoma.

for carcinoma of the breast. There were 79 patients in whom there was histological involvement of both the axillary and internal mammary lymph nodes. Of these, 71 have died of the disease, 3 are alive with disease, and 5 are alive and well after 10 or more years without evidence of recurrence (6·33%). In the present series, 3 of 7 patients are alive and well at 10 or more years without evidence of recurrence (42·8%). The standard error of the difference between the proportions is 18·9%. The observed difference is 35·47%, 1·88 times the standard error, and therefore not quite significant at 95% level of confidence.

These 7 cases can be further analysed on the degree of axillary lymph node involvement, and on histological grading, which was kindly performed by Dr. John Arthur without knowledge of the clinical features. In 3 patients there was extensive axillary node involvement (cases 3, 6, and 11). All have died of the disease although one survived 9½ years (case 3). In the other 4 patients axillary node involvement was limited to 3 nodes or less, 3 of these patients are alive and well at 11 to 12 years (case 7, 8 and 10) and the fourth died at 11 years (case 12). There was no obvious relation between histological grading and outcome (see Table I), best shown by the grades of the 3 surviving patients (high, intermediate and low).

The results of hormonal surgery bore no relation to the results of urinary oestrogen estimations. Of the 4 patients studied, 3 did not derive any benefit from the operation (cases, 1, 2, and 4), whereas one survived 9½ years (case 3). Post-operative urinary oestrogen levels were low in all the patients studied, the highest being found in the patient with the longest survival. It seems unlikely that poor response to operation is the result of persisting oestrogen secretion.

The 5 women who survived for prolonged periods without recurrence appeared to have full and active lives. This certainly applied to patients 3 and 12, who died 9 and 11 years after operation. The terminal stages were short—about 1 month in one and 6 in the other. The 3 patients still alive are also able to carry out their usual duties as housewives, although patient number 10 had had to have a big toe amputated for a melanoma, number 8 feels a little unsure of her back following the adrenalectomy incisions, and number 7, an anxious individual, states that she has not felt the same since the operation, although she is generally very active.

In assessing the quality of life in these patients, the effects of bilateral oophorectomy on sex function have to be considered, but many of the patients were near, or past the menopause when the operations were performed. Of the younger ones, numbers 1 and 7 led active sex lives after operation. In patient 4, survival was brief, and no information on this point was obtained. Of the older patients with prolonged survival, no information is available on patient 3. Patient 8 reported that her sex life had not been altered by the operation, but that it had never been very active. Patient 10 has found that libido is reduced, and patient 12 was a widow.

It is not possible to derive any definite conclusions from this limited pilot study. The results suggest that early oophorectomy and adrenalectomy may improve the expectation of life in women who have carcinoma of the breast with spread confined to the axillary and internal mammary lymph nodes. However, the improvement did not reach the level of statistical significance. If the disease has spread beyond the primary lymphatic drainage area, hormonal surgery in absence of symptoms cannot be recommended. It can also be affirmed that women who have undergone oophorectomy and adrenalectomy are able to lead full and active lives.

We wish to thank Mr. R. S. Handley for allowing us to include his results as our " control " series, Dr. John Arthur for his histological assessments and Dr. Bulbrook and Dr. Greenwood for performing the oestrogen assays and allowing us to include them.

### REFERENCES

Brown, J. B., Bulbrook, R. D. and Greenwood, F. C.—(1957) *J. Endocr.*, **16**, 49.
Handley, R. S., Patey, D. H. and Hand, B. H.—(1956) *Lancet*, i, 457.
Nabarro, J. D. N.—(1960) *Br. med. J.*, ii, 553.
Patey, D. H.—(1960) *Br. J. Cancer*, **14**, 457.
Patey, D. H. and Dyson, W. H.—(1948) *Br. J. Cancer*, **2**, 7.